# Automated code development based on genetic programming in graphical programming language: A pilot study

**DOI:** 10.1371/journal.pone.0299456

**Published:** 2024-03-07

**Authors:** Pavel Kodytek, Alexandra Bodzas, Jan Zidek

**Affiliations:** Department of Cybernetics and Biomedical Engineering, VSB-Technical University of Ostrava, Ostrava, Czech Republic; Wroclaw University of Science and Technology: Politechnika Wroclawska, POLAND

## Abstract

Continual technological advances associated with the recent automation revolution have tremendously increased the impact of computer technology in the industry. Software development and testing are time-consuming processes, and the current market faces a lack of specialized experts. Introducing automation to this field could, therefore, improve software engineers’ common workflow and decrease the time to market. Even though many code-generating algorithms have been proposed in textual-based programming languages, to the best of the authors’ knowledge, none of the studies deals with the implementation of such algorithms in graphical programming environments, especially LabVIEW. Due to this fact, the main goal of this study is to conduct a proof-of-concept for a requirement-based automated code-developing system within the graphical programming environment LabVIEW. The proposed framework was evaluated on four basic benchmark problems, encompassing a string model, a numeric model, a boolean model and a mixed-type problem model, which covers fundamental programming scenarios. In all tested cases, the algorithm demonstrated an ability to create satisfying functional and errorless solutions that met all user-defined requirements. Even though the generated programs were burdened with redundant objects and were much more complex compared to programmer-developed codes, this fact has no effect on the code’s execution speed or accuracy. Based on the achieved results, we can conclude that this pilot study not only proved the feasibility and viability of the proposed concept, but also showed promising results in solving linear and binary programming tasks. Furthermore, the results revealed that with further research, this poorly explored field could become a powerful tool not only for application developers but also for non-programmers and low-skilled users.

## Introduction

Graphical programming refers to a category of programming languages that use visual representations, such as icons, symbols, diagrams, or other graphical elements, to facilitate the design and creation of software applications. Unlike traditional text-based programming languages, where the code, i.e., textual commands, are written in text editors or integrated development environments, graphical programming allows users to interactively create programs by manipulating and connecting graphical elements. Since graphical programming does not require a strong understanding of the language and its syntax, these languages are often designed to make programming more intuitive and accessible to non-programmers.

Automated code development in LabVIEW or any other graphical programming environment is inspired by reversing a standard software development model. This engineering design process can be perceived as a methodical series of steps that allow programmers to create functional products and processes [1]. This process can be highly repetitive, and certain stages often require multiple iterations before proceeding to the next step. Since requirements-based testing and validation, also known as test-driven development, is a common and essential part of software development [2] and a standard procedure for programmers who must verify the code’s functionality, by reversing this process, we can automatically generate code instead of developing programs or unit testing frameworks. In this reverse scenario, we can automatically create programs based on the predefined input requirements, and by backpropagating the input- output differences, we can modify the generated code until all requirements are satisfied. By transforming this task into a fully automated process, we can therefore fundamentally reshape the development principles for basic programs, and instead of employing human experts for code development and test report validation, we can utilize computers to generate programs and evaluate test reports.

Automated code generation in textual-based environments has been used in the software industry for decades [3], and especially in recent years, many novel program generation approaches have been proposed and evaluated on common benchmark problems [4]. These approaches to code generation employ various techniques, including artificial intelligence, machine learning, or genetic evolution methods, to repair or generate efficient and error-free codes. A significant research direction in this field involves the use of machine learning, especially neural network models. Most of these studies employed recurrent neural networks [5–7], transformer models [8,9], or convolutional neural networks [10,11], which are able to learn patterns and structures from given code samples. By training these models on large code repositories, they can capture syntax, semantics, and even higher-level programming constructs, which enables them to generate usable code [12].

Another popular direction for automated code generation includes evolutionary algorithms, particularly genetic algorithms [13] and genetic programming. Genetic programming (GP) is technically regarded as a special evolutionary algorithm inspired by Darwin’s evolutionary theory, where algorithms are characterized by the existence of a population of individuals exposed to various environmental circumstances that lead to natural selection. [14] Unlike genetic algorithms, in genetic programming, the individuals in the population are computational programs, which are typically represented as sequences of instructions or expression trees. [15] These populations are iteratively transformed and evolved over generations into other populations by applying genetic operations to aproximate or find a solution to a specific problem. [16] To measure the degree of adaptation of individuals to the environment, usually a fitness function is employed [16]. However, also other alternative measures could be utilized, for example, Wasserstein distance [17] for probability distribution outputs, or Single-valued Neutrosophic Cross-Entropy [18], which measures the dissimilarity in cases of uncertain or incomplete information. This approach to code generation is primarily useful for optimizing codes for specific tasks or constraints and usually require user-defined input/output examples. However, studies using combinations of input/output examples with natural language descriptions can also be found [19]. One of the biggest advantages of using genetic algorithms based on user-defined requirements, which is the primary focus of this study, is the potential usage of such a system by non-programmers. A well-known case of such a program is, for example, Flash Fill, which is one of the most used data tools integrated into Microsoft Excel that is able to automatically fill out the data in sheets by using predictive technology. [20] According to the comprehensive survey on program synthesis with evolutionary algorithms conducted by Sobania et al., the most frequently used approaches for code generation involve stack-based GP, using mostly Push as a representation language, grammar-guided GP (including tree-based and linearized grammar-based approaches), and linear GP [21] Although the stack-based GP approach makes up the largest proportion of the identified studies (37 in-scope papers), due to its most common language representation (Forth, Push, or Postscript programming language), it is not considered relevant in real-world software projects, especially from the perspective of software development [21]. Other meta-heuristic algorithms introduced in specific fields of automated programming, especially regarding optimization tasks, may include the particle swarm optimizer, gravitational search algorithm, artificial bee colony algorithm, grey wolf optimizer [22], or differential evolution [23] and slime mould algorithm [24,25].

Even though numerous code generation methods have been proposed for textual-based languages in the last few decades [26,27], to the best of the authors’ knowledge, none of these methods have been implemented in graphical-based programming languages, especially LabVIEW. Moreover, the implementation of the actual state-of-the-art methods in a graphical language is rather inefficient and almost impossible without using text-to-object converters since all existing algorithms are primarily designed for text-based languages. Due to the fact that most of the graphical programming environments, including LabVIEW, do not even have such converters or do not support textual compilers, the main aim of this study is to create a proof of concept for a yet unexplored automated GP-based framework for code generation in the graphical programming environment LabVIEW. The proposed code generation framework fully depends on the input requirements, which can be defined even by users without any prior programming knowledge. The entire framework was tested on four basic benchmark problems encompassing fundamental data types, such as string, boolean, numeric, and their combinations. The achieved evaluation results not only demonstrated the algorithm’s ability to generate functional programs in string, numeric and boolean domains, but also proved that the algorithm is able to work in a solution space that isn’t strongly typed, and therefore, can lead to universal solutions. Although the generated solutions showed a significant degree of complexity in comparison to programs written by SW developers, the outcomes of this study prove the feasibility of this idea, where even non-programmers and low-skilled users could efficiently generate programs. The proposed approach in this study is, therefore, the first of its kind in this research field and may serve as a good starting point and inspiration for researchers and programmers working with graphical programming languages. Since graphically oriented programming is recently on the rise with the growing industry 4.0, where PLC-based systems and fast test-developing environments such as LabVIEW or Teststand play a significant role, introducing such algorithms to these environments would help to solve many human-restricted problems.

## Materials and methods

LabVIEW, as a graphical development environment, utilizes a different code representation in comparison to traditional text-based programming languages. This representation involves indexing functions, which cannot be translated directly into human-readable and understandable text as in typical text-based environments. This difference in representation complicates the usage of text-based language prediction models like GPT-3 [28] or other commonly used models for generating code. Hence, in this scenario, genetic programming appears to be the most suitable approach, which evolves and optimizes graphical structures toward user-defined requirements. The following chapters are devoted to the LabVIEW programming environment and its language syntax and provide a detailed description of the proposed code generation framework.

### Labview development environment language syntax

LabVIEW, short for Laboratory Virtual Instrument Engineering Workbench, is a visual programming environment commonly used in measurement, automation, or control applications. Unlike traditional text-based programming languages, LabVIEW utilizes a graphical data flow programming paradigm where the code is represented in the form of interconnected graphical elements called virtual instruments, denoted as VIs, that can be perceived as functions or subroutines in conventional programming languages. The abstract syntax is typically represented as a data flow graph or a block diagram, where each VI or block is a self-contained unit of code or a native function. The execution of the program is then conceptualized as a flow of data, where variables are propagated via the wires through a sequential series of connected functions, which execute as soon as all data is available on the inputs.

**Fig 1:**
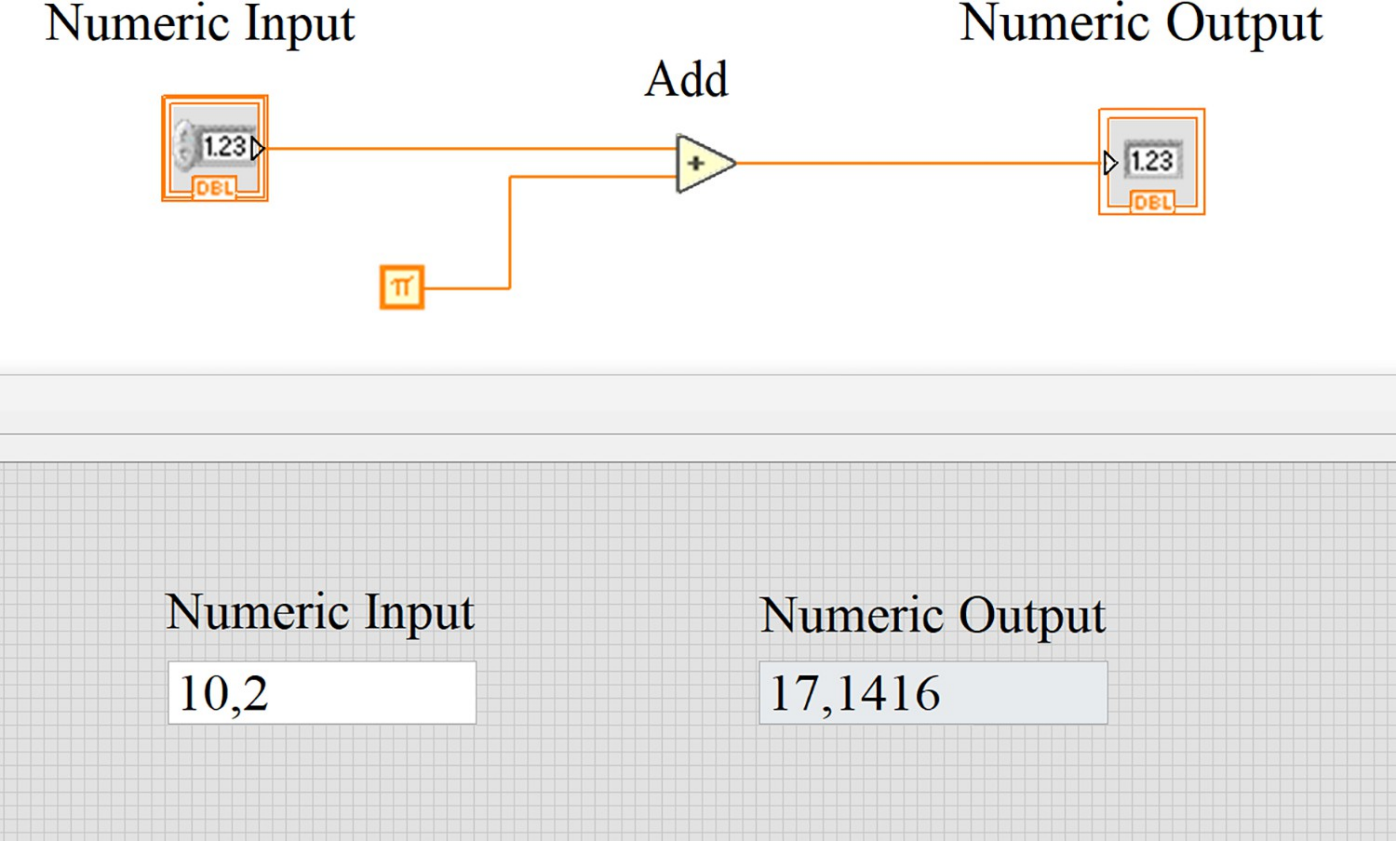
The created front panel and block diagram for a virtual device called "add_pi".

The core of each VI is divided into two interdependent parts: the front panel, which represents the user interface, and the block diagram, i.e., the code, responsible for the program’s logic and functionality. The visual representation of both parts of a simple part of a code is demonstrated in Fig 1.

If we analyze this part of the code from the programmer’s viewpoint, the created method in LabVIEW carries the name add_pi and has one numeric input as a parameter. The output of this function is then an input value increased by the value of π. However, in a much deeper sense of the language, the created program contains four basic objects (two input objects, a function, and an output), where each object is represented by a specific icon in the block diagram. These objects can be considered instances of objects in object-oriented programming, and therefore each of the four elements contains its own private data and methods (such as captions, labels, or set-value methods). Since every single object inserted in the block diagram is a child of a prime class called LabVIEW Object, each element created in the block diagram and also on the front panel is a child of this class. Hence, if we are able to refer to any object by using a pointer, we can also programmatically change its publicly accessible data or invoke its publicly accessible methods. It is also possible to get this reference through all objects contained in the data of our "main" object, which is our program. Through the reference, we are therefore able to obtain references to all front panel or block diagram objects.

Additionally, apart from the four main objects, there are three secondary objects in the given diagram that are presented as connections or wires between the functions, which ensure the data flow of the program. The main difference between a main object and a secondary object is that a secondary object reference can only be obtained from the main object reference to which the secondary object is assigned (connected). Even though for most LabVIEW programmers, this object is just a simple wire connecting two blocks or functions, it is a sophisticated class that, in its private parameters, stores information about its description, the connected terminals, state, program pauses, connection points, or references to the main objects. Moreover, this class allows navigating the program from one place to another by using a set of obtained references to various block diagrams or front panel objects.

Although most programmers do not use this information and do not need to understand these concepts, it is important knowledge that allows performing automated program development tasks in the LabVIEW programming environment.

#### LabVIEW scripting

An essential LabVIEW feature that was used in this work is a LabVIEW VI Scripting software add-on, which provides a set of functions used to access advanced private methods and information that is normally not available to the user. This includes functions that are used to perform code analysis, editing, or even code creation. Nowadays, many leading developers use these features to create templates or automatically generate frameworks for other developers; however, this work deals only with methods allowing to programmatically insert and connect objects within the block diagram. The particular LabVIEW scripting processes employed in this work are depicted in Table 1.

**Table 1:** Basic types of LabVIEW scripting functions used for the purpose of automated code generation.

Process Type	Detail	Function
Navigation	Between function and wire	Trace dependencies and connections from the function to the wire
	Between wire and function	Trace dependencies and connections from the wire to the function
	New VI	Creates (insert) new method/function
Creation	Object on a front panel/block diagram	Creates (insert) new objects (native LV functions, controls) in the program
	Wire	Creates (insert) connections between functions
	Object positioning	Changes the position of objects inside a block diagram

As it might be seen, scripting is a diverse and powerful tool for modifying the final program, and by combining these functions with built-in LabVIEW functions responsible for the run of the program, assignment of the values to inputs, and their reading or evaluation, we can obtain a tool that is an essential part of automatic program development, and which enables us to create or modify the particular programs.

### The proposed framework

**Fig 2:**
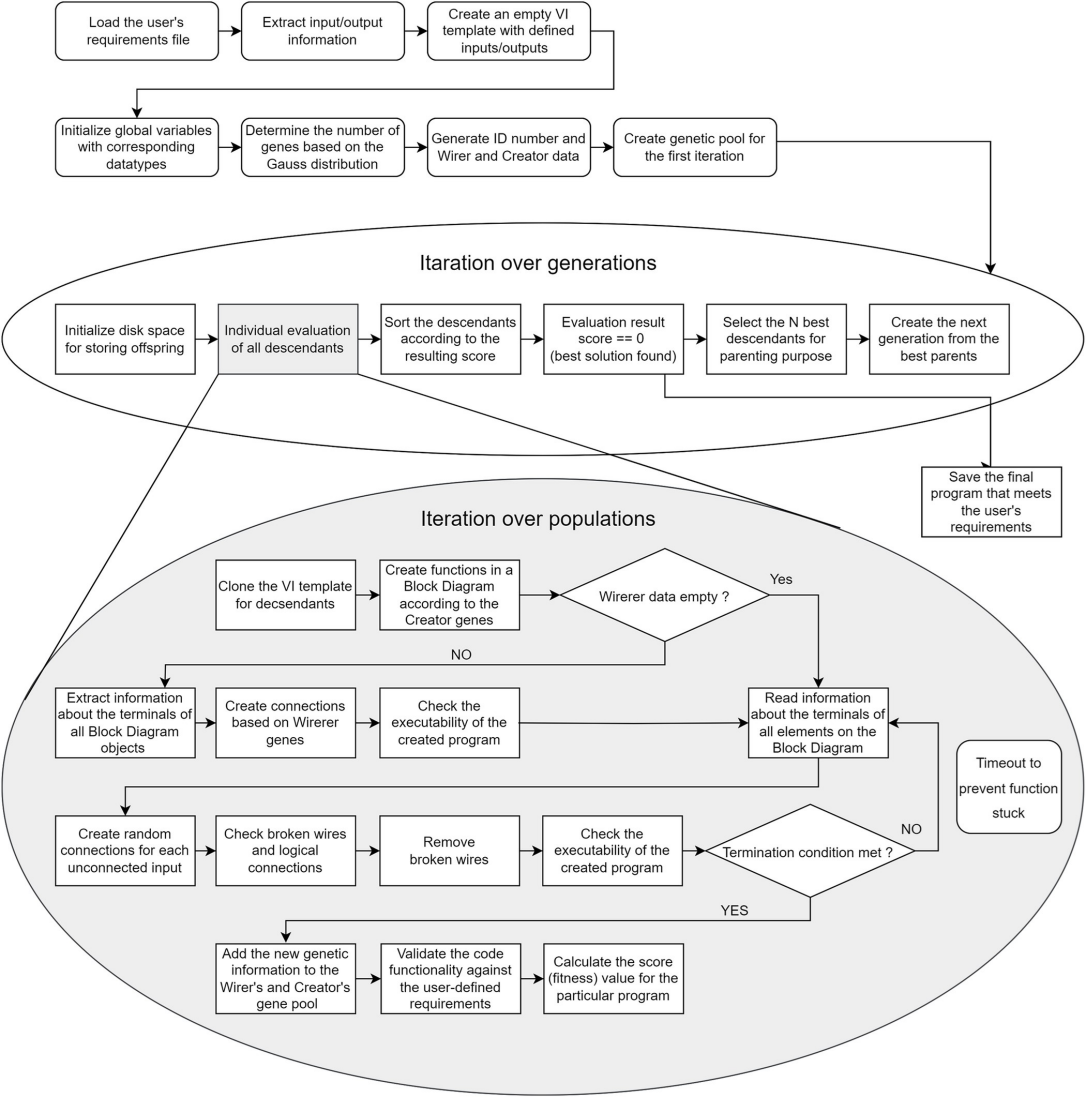
The entire sequence of the proposed code generation framework.

In this study, we approach the problem of automated code development in a similar way as human evolution works. Each generated VI, which is a final representation of a program, can be seen as a human phenotype that represents the complete characteristics of an individual from the generation. Since only the creation and navigation scripting methods are utilized for the code generation, the whole code information can be obtained in two sets, the Wirer and the Creator, which are thoroughly described in the chapter Genetic structure of the program. The process of the proposed code generation approach, from requirement definition to the formation of a new generation is depicted in Fig 2 and described step by step in the subsequent chapters.

#### Genetic structure of the program

**Fig 3:**
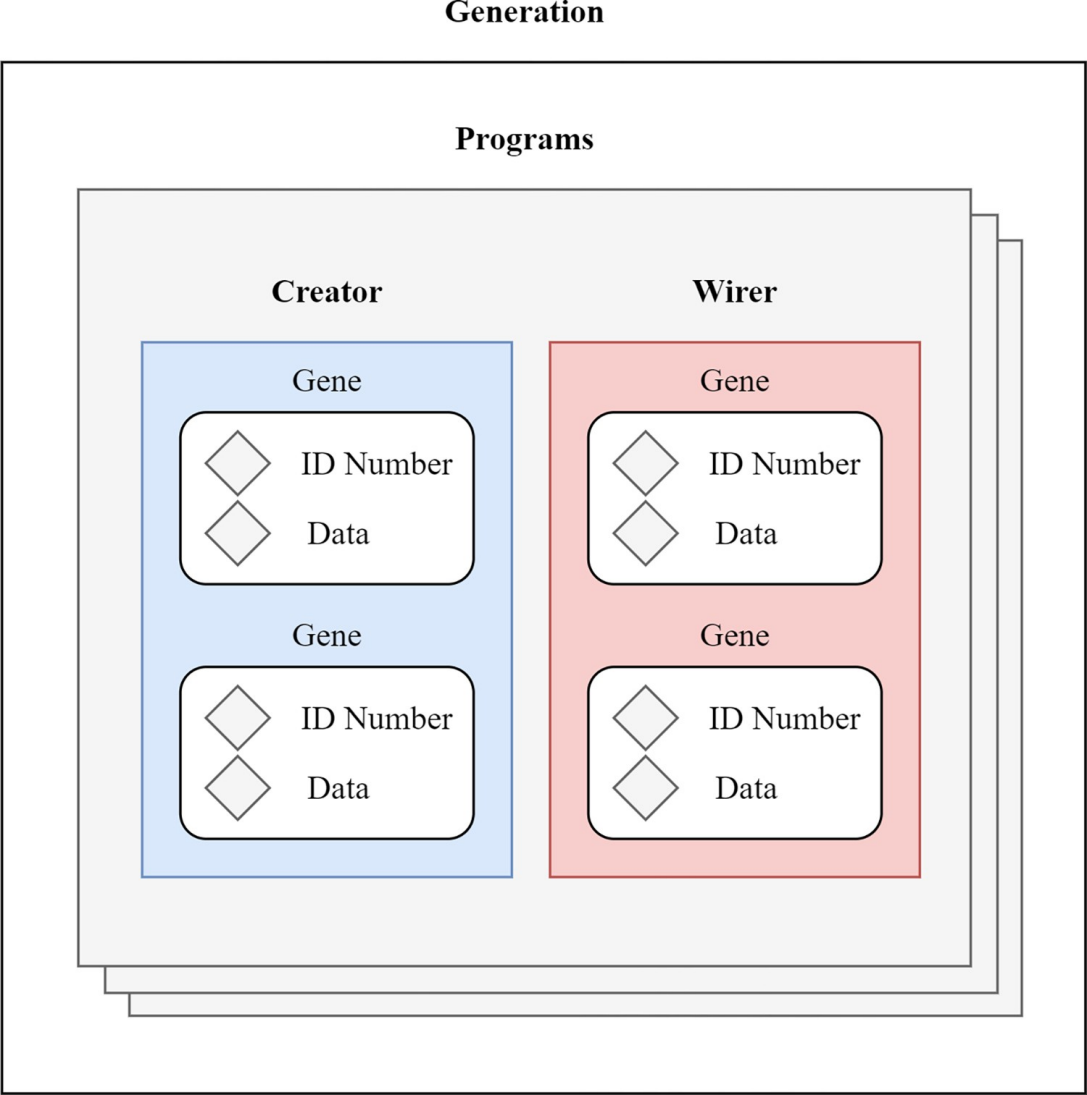
The proposed genetic structure of programs.

The Wirer and the Creator can be perceived as chromosomes in human cells, and just as human chromosomes, the Wirer and the Creator set contain a collection of genes, where each gene, in our case, carries specific information about the structure (the created functions or other objects such as constants) or binding (connections between the program building blocks). Therefore, whereas the creator is responsible for the insertion of functions on the block diagram by using the creation scripting method, the Wirerer is responsible for tracing the functions’ inputs and outputs and for creating connections between the particular functions on the block diagram (performed by utilizing the navigation and creation LV scripting methods). The proposed complete genetic structure is demonstrated in Fig 3.

Similarly, as the individual genes create cell characteristics by using sub-alleles, the genes contained in the Creator and the Wirer create the final form and the behavior of the generated code. Each gene in both sets, therefore, contains an identifier in the form of an unsigned 8-bit integer that is used to assign the gene a specific function (such as a mathematical operation, equal function, or string function) and data in the form of a byte array, which contains all necessary information about the particular element defined by the identifier. For instance, if the gene is mapped as a string constant (defined by the identifier number 0), then the corresponding data within the gene is converted from a byte array back to a string. On the other hand, in the case of a mathematical function (identifier 3), only the first byte of the data carries information about the pointer to a specific numeric function, such as addition (value 0), subtraction (value 1), etc. A detailed explanation of how genetic identifiers are mapped into specific functions is provided in Chapter Initialization process, Table 4. By employing byte array data representation for the data within the Wirer and the Creator, we can furthermore meet the possible requirements for the infinite number of genes in both sets, which ensures no limitations by unnecessary conditions during the program development.

Although the Wirerer contains the same genetic structure as a creator, its only function is to create connections between the existing functions. To connect an output of one function with an unconnected input of another function, the navigation LabVIEW scripting method only requires references to the corresponding input and output terminals of the involved functions. Due to this fact, the Wirer does not use the information obtained in the identifier, and only the connector’s information is extracted from the data, where the first and second elements of a byte array are converted to an integer value representing the indexes of the corresponding output and input terminals.

#### Definition of system requirements

To ensure that the system functions in accordance with the abovementioned goals, it is necessary for the user to have the possibility of defining the input and output variables of the system. According to the requirement definition, a fundamental requirement for our system was to maintain human readability and be easy to understand so that even non-programmers could generate codes. The proposed system in this work supports four basic data types, namely boolean (Bool), string (Str), double (Dbl), and integer (Int). All the user’s functionality requirements are stored in the form of an array of elements, where each element is, like each gene, represented by a control name and an array of values for a given parameter. These parameters are then, at the beginning, loaded into the system and stored in a functional global variable (a frequently used LabVIEW design pattern allowing controlled access to data) so they can be reused in the evaluation process. Since we used a byte array for storing the data within the genes, there is no need to address the issue of different functions’ data type compatibility requirements at this program level. The evaluation function then accepts an array of inputs and outputs.

The input interface for a common user is realized by using the freely accessible library CLAUDIE_XLSX (Compact Library and Universal Data Import Export xlsx), which allows writing and reading data from Microsoft Excel [29]. The file structure was selected in a way that facilitates the user’s ability to enter his requests in the form of input combinations and their required outputs. An example of an implemented structure for a specific task is demonstrated in Table 2.

**Table 2:** Input values example for a numerical problem.

in	in	out
dbl	dbl	Dbl
Num_1	Num_2	Num_Ind
5	10	15
6	11	7
15	42	57
17	43	60
36	44	80

The first file line defines whether the element is an input (control) or an output (indicator), the second line defines its data type (bool, dbl, int, str), the third line declares the name of the element and the following lines create the inputs together with the desired output values.

#### Initialization process

The initialization of default parameters is a critical and challenging problem in evolutionary algorithms. Within this step, the algorithm creates the initial population by generating individuals with constrained random parameters for the subsequent evolutionary process. All population parameters with particular value ranges are depicted in Table 3.

**Table 3:** The used population parameters with the corresponding parameter values.

Parameter	Value
Population size	Fixed value
Complexity	Randomized with Gaussian distribution–mean selected and sigma 0.1
Identifier	Randomized in a value range of 0–8 according to Table 4 with a uniform distribution
Data within the Creator	Generated byte array of U8 values with uniform distribution.
Data within the Wirerer	Two random U8 values with a uniform distribution in an array

Since it is usually not very common to control the size of the population [30], this parameter is in this work set to be optional for the users, and for all performed tests, it was set to a constant value, usually 1000. The second adjustable population parameter used in this study is the complexity parameter (set in the range from 3 to 20, depending on the problem’s complexity). This number represents the mean value of a Gaussian curve, which is formed by the number of genes generated for each descendant in a population. In other words, the number of genes in a generation likely corresponds to a Gaussian curve with a defined standard deviation of 0.1 and a mean equal to the selected complexity value. This "randomness" ensures a better distribution of the generated code possibilities already in the first iteration and prevents the algorithm from getting stuck at the local minimum [31].

To assign the gene a LabVIEW object (function, control, or a constant), we proposed a mapping table (refer to Table 4), which takes a randomly generated number of an identifier in a defined range and, based on its value, assigns the identifier a specific LabVIEW building block represented by a native LabVIEW ID class number. These LabVIEW ID numbers can be perceived as inner environment identifiers for particular parent classes, where each LabVIEW building block belongs to a specific parent class. Even though the knowledge of the predefined LabVIEW classes is not required for common programming tasks, it is part of the basic concept of the environment.

**Table 4:** An overview of the used identifiers and their relation to specific block diagram objects and data usage.

Gene identificator	Num ID in LV	Building block/Object	Data usage
0	16392	String Constant	Converts byte array to string
1	16395	Control Terminal	Gene data not used
2	16476	Bundler	Gene data not used
3	16400	Math. Function	Maps the first byte of the data array to a mathematic function
4	16390	Digital Numeric Constant	Converts byte array to a number
5	16422	Boolean constant	Gene data not used
6	16400_1	Bool Function	Maps the first byte of the data array to a boolean function
7	16429	Equal Function	Gene data not used
8	16400_2	Select Function	Gene data not used

The problem that we encountered with this LabVIEW inner categorization was a multiple occurrence of the class with an identifier of 16400 for significantly different functions. Even though the parent is identical for different descendants, such as mathematical functions, boolean functions, and the function select, which is our case, these functions do not have the same inputs, outputs, or meaning. To solve this problem, we divided these descendants into individual subclasses. This means that the gene identifier doesn’t contain the inner LabVIEW class number but instead contains an extended unique identifier that differentiates the problematic classes. The following Table 4 shows the relation between the identification numbers and the corresponding LabVIEW functions and explains the usage of the gene data for the particular identifiers.

Another concept that we employed during the initialization process in this work is strongly typed genetic evolution. Unlike classical programming, where any function can be inserted into the code, STGE limits the number of available functions. This means that only functions with a corresponding data type to the selected data type are available. An exception is a boolean data type, where, for instance, the "equal" function is a dynamic function that can be used by all data types. It is important to note that since this approach reduces the available functions, the mapping table (Table 4) dynamically changes according to the allowed data types. Introducing this procedure directly affects the values that can be written into the gene’s identifiers, and so by limiting these values, STGE helps speed up the evolution process and significantly increases the chances for a successful evolution [32].

#### Wirer and Creator data generation

Simultaneously with the generation of the identifiers for the Wirer and Creator sets, another parallel process produces, for each created identifier, gene data in the form of a U8-byte array. For the generation of the data within Wirer genes, we applied an initialization rule where two numbers from the range of <0, Gauss (complexity; 0,1)> were selected on the basis of the uniform probability. This rule ensures that each element of the program has an equal chance of being joined with any other element. These generated numbers are then inserted into a U8-byte array that represents the particular gene data. On the other hand, for the data in the Creator, we had to consider all the possibilities that may arise on a theoretical level. Since the Creator assigns information (the generated data) to a created function on the basis of the function’s identifier, there is a probability of assigning data to functions that do not need that information, such as select or equal functions (refer to Table 4 for functions where gene data are not applicable). In a practical application, this case doesn’t seem to be a big problem, but in a worst-case scenario, the Creator might assign data to a string constant where the user is expecting a specific input (the infinite monkey theorem). To prevent this occurrence, the algorithm for generating Creator data generates a sequence of numbers in a range of 0–255 based on an even distribution of probabilities until the algorithm meets the defined stopping condition. Unlike in the case of the Wirer, this process, therefore, continues to a potential infinity as long as another randomly generated number from the interval <0; 1> is greater than 0.95*i*, where *i* is the length of the currently generated string. This approach theoretically enables infinitely large text and ensures maximal variability.

#### Creation of new child

The creation of a new child from the individual Creator and Wirer genes is implemented in this work sequentially, where each gene is processed separately. At first, all genes from the Creator are processed, which means that all program building blocks, i.e., functions or constants, are created based on the gene identifier and data by calling the LV scripting creation function. All created functions are, during this process, placed on a block diagram at a random place, so the current code doesn’t have to meet the programming standards yet (clean code without unnecessary bends in block diagram wires, top-down and left-right data flow layout, and many more). In the case of block diagram constants, the algorithm also handles empty gene data by generating a random constant value. Although this case doesn’t apply to the initialization phase, during the evolution, the genes might lose the genetic material within the data. After creating all the building blocks on the block diagram, the algorithm processes the Wirer genes and creates links between the block diagram functions, i.e., between the output of the first object and the input of the second object. During this process, the algorithm extracts all objects’ input and output terminals by using the LabVIEW scripting navigation method, and from the extracted information (references to terminals), it creates two arrays representing block diagram objects’ inputs and outputs. The final connection of two functions or objects on the block diagram is then realized by creating wires between the indexed elements from the above-mentioned arrays, where the array index numbers are obtained from the gene data. To increase the chance of finding a suitable connection, we complemented this process with a controlled selection, where the whole array of input terminals is filtered based on the output terminal data type. Since in the LabVIEW programming environment it is possible to connect two different terminals with different data types, which results in a broken wire and non-executable programs, by employing this filtration, only input terminals with a corresponding data type to a selected output terminal are preserved.

In the last phase of the process of creating a new child, the generated section of code is evaluated for its functionality. During this evaluation, the program might not pass for several reasons. The main reason for failure is a poor logical interconnection between the functions. This can be caused by unconnected terminals, broken wires, introduced feedback loops (where a function input is linked to the same function output, causing a delay in the output of the execution), or by connecting multiple outputs to one input (which is a problem specific to graphical programming, unlike text-based languages). To eliminate this problem, we implemented an additional sequential algorithm that traces all wires in a block diagram by using a built-in LabVIEW method and then deletes all the incorrectly connected wires. Afterward, for all unconnected user-defined inputs, the algorithm establishes a random connection between a particular input and an existing output with a corresponding data type. Additionally, in the case of any missing required function input, the algorithm creates an appropriate constant with a corresponding data type and connects the constant to a desired input. This process ensures a higher success rate for creating valid and executable code and improves the chances of algorithm convergence.

#### Evaluation of child

A key component that significantly improved the algorithm’s results was the implementation of a back-analysis of the created child. Due to the fact that the above-mentioned process generates random connections and possible constants, it was essential to store this information in a gene pool of the Wirerer and the Creator. Storing this information in the form of newly created genes primarily prevented the descendants with the best results from the development deterioration. The proposed back-analysis algorithm validates the functionality of the particular code samples against the user-defined requirements and is based on the following formula:

$$Err=\sum_{i=0}^{inf}P({\bar{x}}_{i})-S(\bar{x_{i}},W,C)$$ (1)

where the resulting error value *Err* is the sum of all partial differences between the desired value (output) *P* for the selected combination of inputs and the obtained values S (actual outputs), dependent on the values of *W* and *C* representing the sets Wirer and Creator.

Although this task is not challenging according to the development, the performance of this process is the most time-consuming. Since the program’s user-defined inputs and outputs are in the form of arrays, the evaluation process has to be repeated for each child as many times as many input combinations the user defined. Within the evaluation, we implemented a different evaluation logic for each data type in the program. Operations working with a boolean data type result in a "binary" value of a deviation, where 0 stands for a case of a match (T = T, F = F), and the value of 1 indicates a mismatch. On the other hand, the resulting deviation in numeric data type is equal to the absolute value of the difference between the input and output values. Text strings use an equivalent logic, but the deviation is calculated as the sum of all partial results for each ASCII character converted to a U8 data type. All error results for each descendant are then stored in an array for further processing.

#### Creation of a new generation

At the beginning of the evolutionary process, we have to select suitable parents for the new descendants. This task is achieved by sorting the evaluation results by the smallest number and selecting the best available results with the lowest score, i.e., the lowest difference between the achieved and the desired output. Descendants with the best results then become parents. The number of selected descendants for the next evolution was determined by the trial-and-error process and, for the majority of the experiments, was set to 6. To avoid generating similar local maxima due to identical evaluation results, only the first occurrence of the duplicate values is selected. In this study, one-to-one inheritance was employed, so each child has exactly one parent [33].

**Fig 4:**
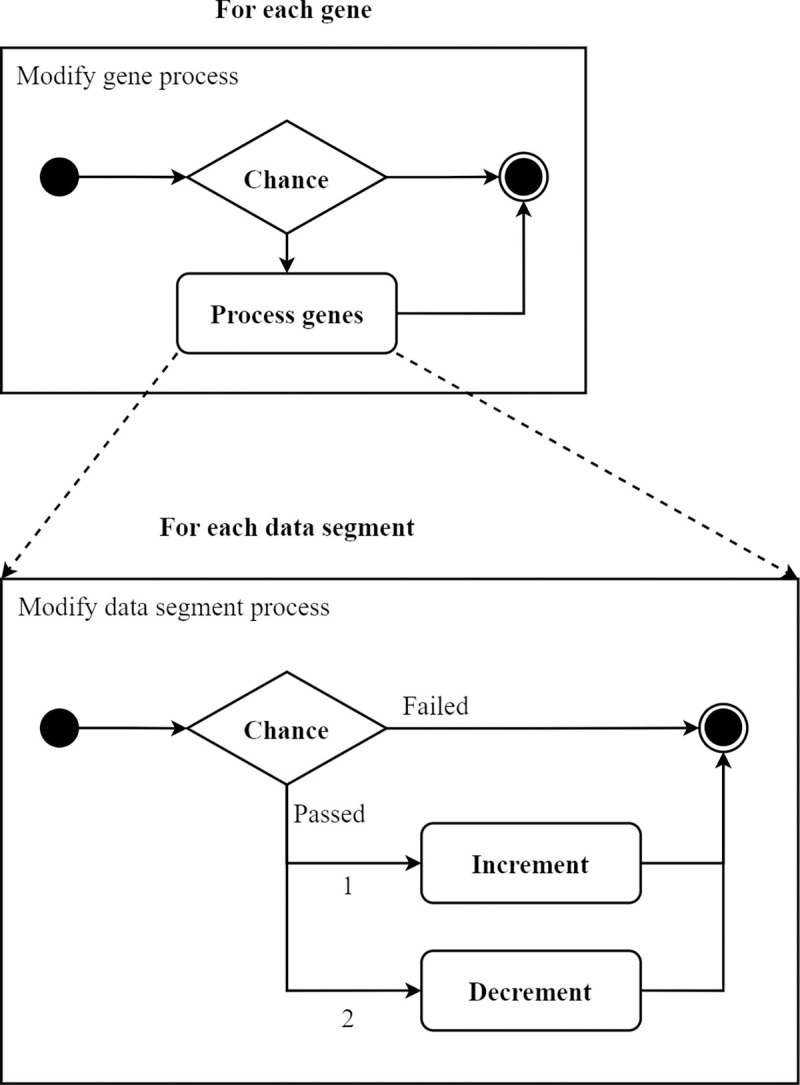
The sequence of processing genes during the evolution.

After selecting the best parents, the algorithms finally proceed to mutation-based evolution by applying mutation to the Creator and Wirer genes. In the case of the mutation of Creator genes, a random value from a range of <0, 100> is generated for each gene in the set. The individual genes are then modified only if the generated value is less than 25. This modification applies to the identifier, which affects the change of the function, as well as to partial values in the gene data (every single element in the byte array). In the case of the gene modification with a 1/4 probability, there is an additional 25% chance that the current identifier or data element value is decreased by a randomly generated number in a range of 1–3, a 25% chance that the current value is increased by the same number, and a 50% chance that the particular gene value is not changed. The overall probability of changing the gene within the Creator set is, therefore, 12.5%. A similar logic applies also to the evolution of the Wirerer genes, only with different modification probability values. The Wirer genes, in case of a mutation (also a 25% probability), are in one-third of cases incremented by a number from 1 to 100% of their original value; in 1/3 of cases, the genes are decreased by this number, and there is the same probability that the genes are not changed. The total probability of the mutation of Wirer genes then amounts to approximately 16.6%. This approach to mutation within the Wirer genes ensured higher variability and induced sufficiently large changes in values. Since the Wirer does not use the information obtained in the identifier, this operation affects only the gene data elements, specifically the output and input indexes. The whole process of evolution is depicted in a diagram in Fig 4.

The mutation of genes within the Wirer and Creator is followed by the potential creation and elimination of individual genes, which turned out to be a key feature of a functional code-generating system. While the above-mentioned steps completely imitate the human process of mutation (the shrink mutation operator), the creation and elimination of genes slightly deviate from human evolution. Even though we can observe this process in humans, its manifestation is rather physiological (the creation of a new phenotype by combining the changed alleles). On the other hand, the process of code creation is about creating or destroying an existing gene, which prevents future generations from degradation. The elimination process is performed with a probability of 15% for each gene and results in the removal of a particular gene from the gene pool of the specific set. The same probability is also assigned to the process of creating a new gene, in which a full new gene is added to the gene pool of existing genes. This step is performed for both the Wirerer and Creator genes.

## Experimental evaluation

To verify the proposed framework, we formulated some basic tasks and benchmark problems. Since the proposed study is designed as a pilot study mainly focused on the feasibility of code generation in a graphical programming environment, the proposed evaluation consists of only simple benchmark problems, dealing mostly with linear and binary tasks. Although there are many benchmark problems available for this purpose, all of them introduce loops and cycles that are not included in this pilot study. Due to this reason, we have chosen simple benchmark problems that do not require more complex loops or cycles. The employment of simpler tasks furthermore allowed us to focus more on the performance evaluation of our algorithms instead of analyzing problems arising from the code’s complexity. Within the evaluation procedure, we defined three main prototype problems, including a string model, a numeric model, and a boolean model.

The string prototype problem verification was performed by selecting a function with one input and one output. The particular values for this task are given in Table 5. The desired result of the algorithm is then the addition of a string "ms" to the input value in a numeric format (string formatting). This task is relatively challenging from the perspective of genetic evolution and model testing since the function for concatenating strings has to be evolved, and a string constant with an expected value has to be created at the same time. As we mentioned in previous chapters, the user-defined string can be of any size, and thus it is necessary to mutate and evolve the creation, as well as the destruction of the genes within the data.

**Table 5:** Input and output values for a string prototype problem.

Input Value	Required (Output) Values
100	100 ms
10	10 ms
1	1 ms
500	500 ms

The second prototype problem refers to a numeric problem and represents a multi-input task where the required output is the creation of a mathematical function between the two inputs. Both the selected input values and the expected output values are listed in Table 6. The most interesting task was the addition of the inputs; hence, this problem was chosen as a typical task of the system.

**Table 6:** Input and output values for the numeric prototype problem.

Input Value 1	Input Value 2	Required output
5	10	15
6	11	17
15	42	57
17	43	60
36	44	80

The last prototype problem we defined in this study is the boolean model with two boolean input variables and one boolean output. Within this model, we employed two boolean tasks, including a logical function OR and an EQUAL function. The assignment for the selected task is depicted in Table 7. In this case, the main point was to examine the evolution with a limited ability to verify the outcomes.

**Table 7:** Input and output values for the boolean prototype problem.

Input Value 1	Input Value 2	Required output (Task OR)	Required output (Task EQUAL)
F	F	F	T
T	F	T	F
F	T	T	F
T	T	T	T

## Results

**Fig 5:**
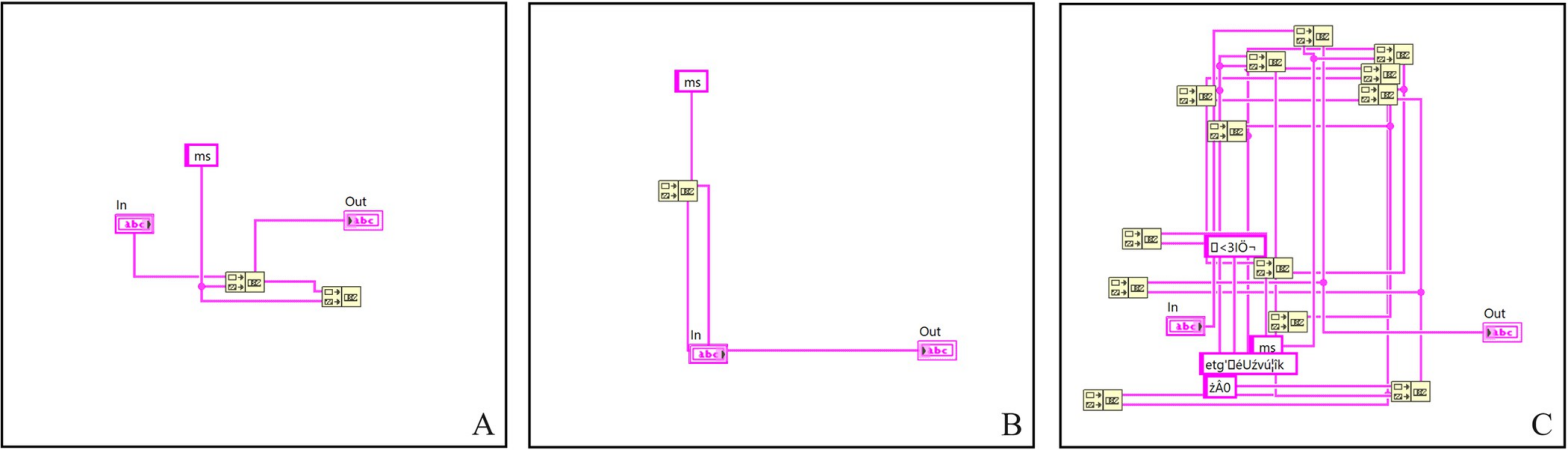
Final solutions of a string problem for multiple algorithms runs.

The first problem, which was evaluated to confirm the algorithm’s functionality, was a string problem. The proposed test model involves two problematic key points, which are related to the conversion of the values inside a data element. This process requires size adjustments as well as the evolution of the values within the data. The results of multiple algorithm runs are shown in Fig 5.

From these results, we can deduce that we can achieve repeatable solutions even with changes in input parameters. The generated results clearly show the ability of the algorithm to find the "optimal" program. In solution (A), the generated code is burdened with an extra function. Nonetheless, this doesn’t influence the code functionality, and the unused parts are removed during the program compilation. In the case of solution (C), we changed the maximum complexity input parameter. This parameter was set for the basic operations in a range of <0; 10> due to higher computational complexity, but in the case of C, we set this parameter to a value of 30. It is apparent that the resulting solution is already out of the optimal and readable code; however, the solution still meets the user’s requirements.

**Fig 6:**
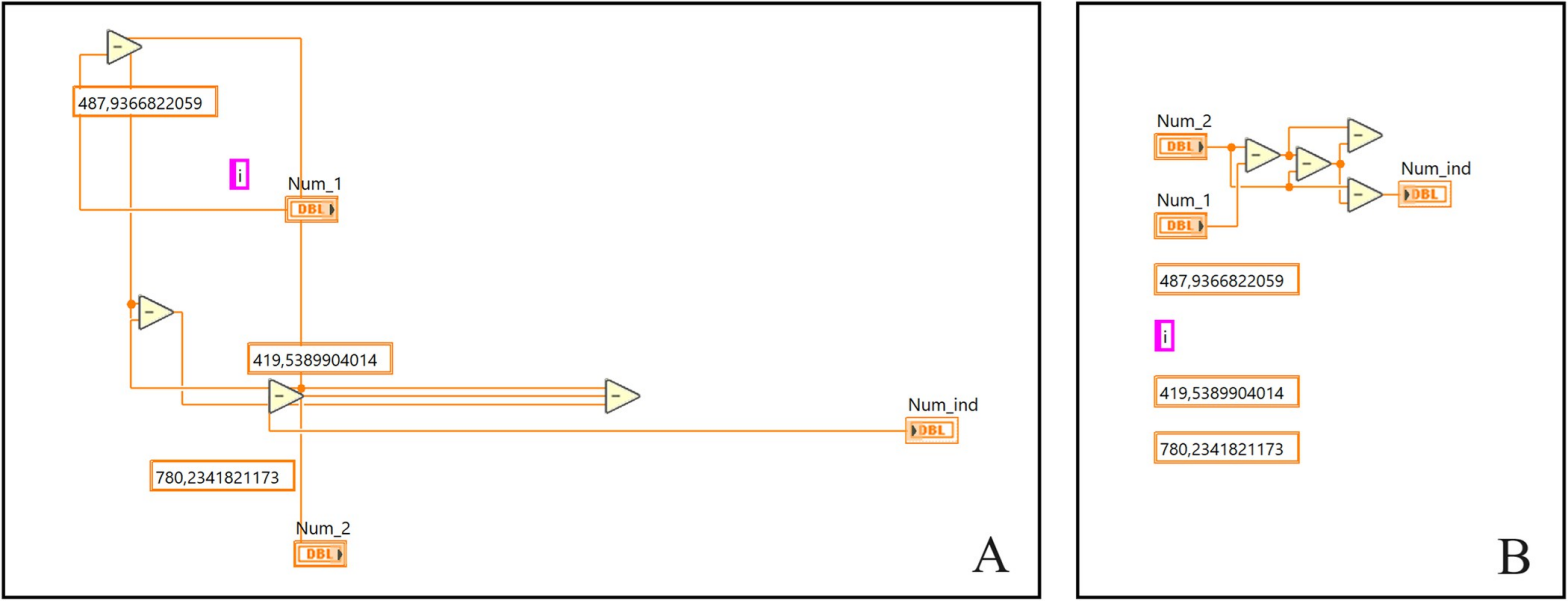
Numeric problem solution. The original form of the generated code (A), the cleaned-up form of the result (B).

A key element during the evaluation of the program’s evolution was the verification of simple addition function behavior. Generated solutions, i.e., programs, during the experimental verification led to clear conclusions and findings that weren’t revealed in more complex mathematical functions. The results of this process are demonstrated in Fig 6.

The first problem that can be observed in this solution is the occurrence of different data types in the code, caused by the disabled usage of strict data types. In the first generation, the program included many more objects that were related to the boolean or string data type, but with subsequent generations, the number of these elements decreased until only elements related to the required data type remained. This was caused by the fact that redundant units of code (creator genes) are strongly dominated by useful functions. Genetic evolution, therefore, leads to a selection of genes that do not include these functions. This effect is not strictly dominant, but a positive trend has been observed. Another curious behavior we noticed was the creation of a function *Y = A + B* by using three subtraction functions that resulted in an equation *Y = A—((A-B) -B)*. This result is considered the correct solution to a problem; however, the solution seems to be extremely complex according to its purpose. This occurrence can be restricted by limiting the complexity parameter.

**Fig 7:**
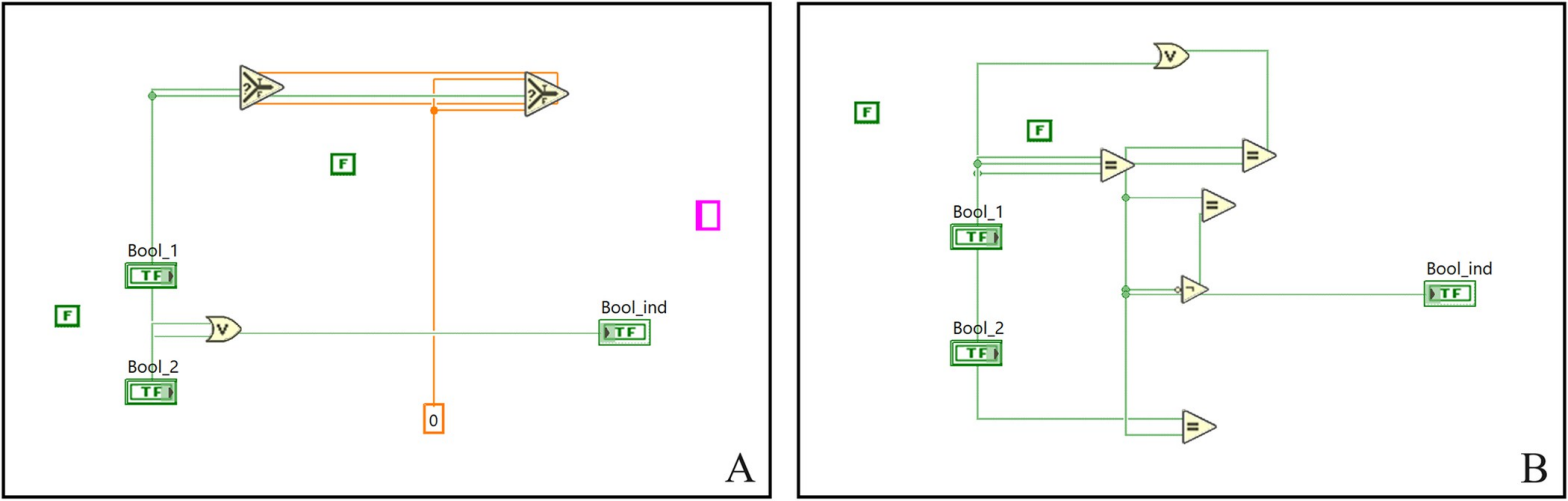
Solution of OR (A) and EQUAL (B) functions of the boolean problem.

In the case of the boolean model, the algorithm was able to achieve satisfying results according to the program’s functionality. As can be seen in Fig 7, due to a higher complexity parameter (a value of 10) and the disabled usage of strict data types, the result of the logical OR function is burdened with a relatively large amount of useless function blocks. On the other hand, for the second test case, including the EQUAL function, we enabled the usage of strict data types while preserving the high complexity parameter. The final solution to this task (B) contains multiple random connections of several equal functions. The result was created within the first generation, where we set a high complexity (10) and enabled strict data types.

The additional last step of the evaluation comprises testing the usage of all basic data types. The output of this task should be a function that converts the input numeric value to a logical value on the basis of the required string output value. The proposed input and output values are listed in Table 8.

**Table 8:** Input and output values for testing the combination of data types.

Input value	Required output
0	Equal to zero
1	Not equal to zero
2	Not equal to zero
3	Not equal to zero

**Fig 8:**
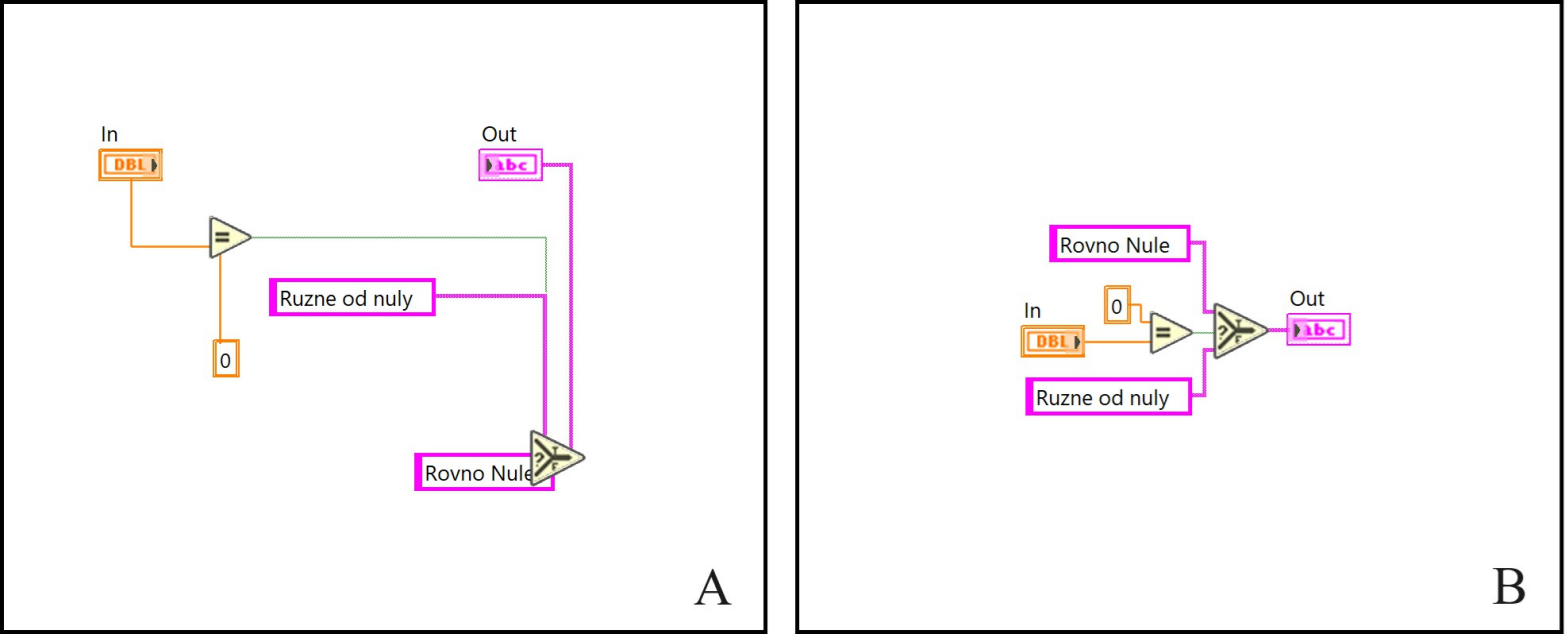
Solution of a combinatory problem. (A) the original code of the solution, (B) cleaned-up solution of the task.

These results (see Fig 8) proved that the proposed algorithm is able to work in a solution space that isn’t strongly typed, and therefore, it can lead to universal solutions. To improve the readability of some codes in this work, we additionally cleaned the code by using a built-in tool that automatically reroutes wires and rearranges block diagram objects. As mentioned before, graphical programming in LabVIEW is based on placing and connecting objects, such as functions, constants, or terminals, on a program’s block diagram. Since during automatic code generation, the user has no control over the functions’ positions or their connections (bends in wires), such automatically generated code does not meet the best coding practices or readability standards, which have to follow the top-to-bottom and left-to-right dataflow paradigm. Therefore, by using the embedded automated cleanup functionality, which is able to adjust the spacing, remove bends in wires, or logically rearrange block diagram objects, the code can meet at least the essential requirements and programming standards.

Another important aspect we analyzed during the technical evaluation was the algorithm’s speed and efficiency. This estimation was realized by measuring the independent processing times of the Creator, Wirer, as well as the evaluation process. To get a good estimation, the processing time for each part was averaged over multiple generations with a population size of 1000 and a set complexity value of 10. The estimated processing times for all phases can be seen in Table 9.

**Table 9:** Average processing times for the individual processes within the evolution.

Tested benchmark model	Creator process duration (ms)	Wirer process duration (ms)	Evaluation process duration (ms)
String	20.08	86.00	19.16
Numeric	19.40	63.28	28.86
Boolean	24.25	85.92	45.20
Mixed type	28.05	62.68	30.29

## Discussion

The main goal of this pilot study was to create a proof-of-concept for an automated code generation approach within the graphical-based programming language LabVIEW. Since, to the best of the authors’ knowledge, none of the automated code generation methods have been implemented in graphical-based programming languages, especially LabVIEW, this study aims to prove the feasibility and practical potential of this proposed concept. For this purpose, we designed and developed a requirement-based automated code generation algorithm that is able to provide functional solutions on the basis of user-defined requirements. These input requirements can be defined by experienced software developers but also by non-programmers or users with little programming experience. The proposed framework in this study was tested on four different benchmark problems that were designed to assess the framework’s ability to generate error-free, functional, and efficient codes across various data types. The proposed string problem model tested the framework’s capability in text manipulation and string operation tasks, the numeric problem model focused on arithmetic operations and handling numerical data, the boolean model dealt with logical conditions and provided insight into the framework’s decision-making processes, and the last mixed-type problem model tested the framework’s versatility in handling multiple data types. The performance of the proposed framework was then evaluated by assessing the generated codes’ accuracy, complexity, execution speed, or adherence to user requirements.

During the experimental evaluation, the designed code generation system achieved success not only in “hill-climbing” tasks, where we were able to find appropriate solutions with a gradient ascent algorithm, but also in one-point search problems, such as the boolean problem. Moreover, the mutation genetic operator, in combination with the proposed approach has been identified as a proper strategy for creating connections in graphical programs. Based on the achieved results, we have to point out that even though the algorithm was, in all tasks, able to find a functional and errorless solution that met all input requirements, these solutions were much more complex and were burdened with redundant objects in comparison to programmer-developed codes. The complexity of the code, in this case, can be interpreted as a higher number of required connected objects that create the final solution. This is mainly caused by the natural behavior of evolutionary algorithms and genetic programming, which focus more on finding the best solution than the optimal solution. Due to this fact, the algorithm proposed in this study might find multiple different solutions that are not optimal, even if they fully satisfy the defined requirements. However, this level of complexity did not affect the accuracy or execution speed of the generated code compared to manually written codes.

Although we are able to demonstrate the satisfactory functionality of the proposed method and prove the feasibility of this concept, we cannot fully declare that the problem is solved. Furthermore, additional research in this field has to be conducted, especially regarding the optimization and finding optimal solutions. Another remarkable fact revealed by the experiments is that a significant portion of the computation time for creating a single child is taken by the Wirer, i.e., by creating interconnections. To optimize this process in the future, it would be beneficial to develop a more sophisticated algorithm that better reflects the actual input and output requirements of the created functions instead of only processing the genetic material in genes and randomizing connections in cases of non-valid solutions. Future research should also be devoted to the implementation of more complex structures, such as cycles or loops, so we can fully exploit all the strengths of the proposed solution and test the methodology on more complex benchmark problems. Moreover, this would allow researchers to compare the proposed approach with existing methods. In addition, exploring multi-parental genetic programming or some types of polygamy-based algorithms can become an important area for future research. Future research should also consider the potential benefits of using cloud computing since the search space could reach enormous dimensions. This would enable researchers to create thousands or even millions of programs in a second, which could be validated and iterated over the best of the best results to find the final solution to much more complex problems.Based on these findings, we can conclude that this pilot study not only proved the feasibility of automated code development in graphically oriented programming languages but also built a strong foundation for further research in this relatively unexplored domain.
