# Correction: Automated code development based on genetic programming in graphical programming language: A pilot study

**DOI:** 10.1371/journal.pone.0302986

**Published:** 2024-04-29

**Authors:** Pavel Kodytek, Alexandra Bodzas, Jan Zidek

The published funding statement is inaccurate. The correct funding statement is as follows: This work was supported in part with the financial support of the European Union under the REFRESH–Research Excellence For REgion Sustainability and High-tech Industries project number CZ.10.03.01/00/22_003/0000048 via the Operational Programme Just Transition. This work was supported in part by the Ministry of Education of the Czech Republic (Project No. SP2024/070).
